# Predicting fracture outcomes from clinical registry data using artificial intelligence supplemented models for evidence-informed treatment (PRAISE) study protocol

**DOI:** 10.1371/journal.pone.0257361

**Published:** 2021-09-23

**Authors:** Joanna F. Dipnall, Richard Page, Lan Du, Matthew Costa, Ronan A. Lyons, Peter Cameron, Richard de Steiger, Raphael Hau, Andrew Bucknill, Andrew Oppy, Elton Edwards, Dinesh Varma, Myong Chol Jung, Belinda J. Gabbe

**Affiliations:** 1 Clinical Registries, School of Public Health and Preventive Medicine, Monash University, Melbourne, Victoria, Australia; 2 Institute for Mental and Physical Health and Clinical Translation, School of Medicine, Deakin University, Geelong, Victoria, Australia; 3 School of Medicine, Deakin University, St. John of God Hospital, University Hospital Geelong, Geelong, Victoria, Australia; 4 Department of Data Science & AI, Faculty of Information Technology, Monash University, Clayton, Victoria, Australia; 5 Oxford Trauma and Emergency Care, Nuffield Department of Orthopaedics, Rheumatology, and Musculoskeletal Sciences, Medical Sciences Division, University of Oxford, Oxford, United Kingdom; 6 Health Data Research UK, Swansea University, Swansea, United Kingdom; 7 National Centre for Population Health and Wellbeing Research, Swansea University, Swansea, United Kingdom; 8 Department of Epidemiology & Preventive Medicine, School of Public Health and Preventive Medicine, Monash University, Melbourne, Victoria, Australia; 9 The Alfred Hospital, Prahran, Victoria, Australia; 10 Department of Surgery, University of Melbourne, Epworth HealthCare, Epworth, Richmond, Victoria, Australia; 11 Eastern Health Clinical School, Monash University, Box Hill, Victoria, Australia; 12 Department of Orthopaedic Surgery, Royal Melbourne Hospital, Melbourne, Victoria, Australia; 13 The University of Melbourne, Melbourne, Victoria, Australia; 14 Epworth Healthcare, Melbourne, Victoria, Australia; 15 Department of Surgery, Monash University, Melbourne, Australia; 16 National Trauma Research Institute, Melbourne, Australia; 17 Department of Radiology, Alfred Hospital, Melbourne, Australia; Public Library of Science, UNITED KINGDOM

## Abstract

**Background:**

Distal radius (wrist) fractures are the second most common fracture admitted to hospital. The anatomical pattern of these types of injuries is diverse, with variation in clinical management, guidelines for management remain inconclusive, and the uptake of findings from clinical trials into routine practice limited. Robust predictive modelling, which considers both the characteristics of the fracture and patient, provides the best opportunity to reduce variation in care and improve patient outcomes. This type of data is housed in unstructured data sources with no particular format or schema. The “Predicting fracture outcomes from clinical Registry data using Artificial Intelligence (AI) Supplemented models for Evidence-informed treatment (PRAISE)” study aims to use AI methods on unstructured data to describe the fracture characteristics and test if using this information improves identification of key fracture characteristics and prediction of patient-reported outcome measures and clinical outcomes following wrist fractures compared to prediction models based on standard registry data.

**Methods and design:**

Adult (16+ years) patients presenting to the emergency department, treated in a short stay unit, or admitted to hospital for >24h for management of a wrist fracture in four Victorian hospitals will be included in this study. The study will use routine registry data from the Victorian Orthopaedic Trauma Outcomes Registry (VOTOR), and electronic medical record (EMR) information (e.g. X-rays, surgical reports, radiology reports, images). A multimodal deep learning fracture reasoning system (DLFRS) will be developed that reasons on EMR information. Machine learning prediction models will test the performance with/without output from the DLFRS.

**Discussion:**

The PRAISE study will establish the use of AI techniques to provide enhanced information about fracture characteristics in people with wrist fractures. Prediction models using AI derived characteristics are expected to provide better prediction of clinical and patient-reported outcomes following distal radius fracture.

## Introduction

Fractures are the most common form of hospitalised trauma, contributing to approximately 200,000 hospitalisations and over one million bed-days in Australia each year [1]. Distal radius fractures are the most common fracture of the upper limb across all age groups [2], second only to hip fracture as the primary reason for admission to hospital for fracture [3]. More than 400,000 people in England were admitted to hospital with a distal radius fracture over a 10-year period [4]. Six percent of women will have sustained a distal radius fracture by 80 years of age [5]. Of concern, the incidence of these fractures is increasing [3, 4]. In the UK, distal radius fracture admissions rose by more than 80% from 2004/05 to 2013/14 [4], while a previous Australian study projected a similar increase to 2021 based on figures from 1997 [6].

Distal radius fractures occur across all ages and are sustained in a range of high (e.g. motor vehicle collision) and low (e.g. fall on an outstretched hand) energy mechanisms and there many operative and non-operative treatment options. Surgery carries inherent risks for the patient and considerable cost implications based on the treatment choice and implant used. While most orthopaedic societies have established distal radius fracture management guidelines, adherence to guidelines is variable, even within a single country [7]. Data from Ireland from 2008 to 2017 showed a significant rise in the rate of plate fixation and a reduction in the use of percutaneous k-wires [8], despite economic analysis showing plate fixation was unlikely to be cost-effective if the fracture can be treated with a closed reduction [9]. In the US, surgical management was found to agree in only 40% of cases where the guidelines recommended nonoperative treatment [10]. A recent meta-analysis of 38 clinical trials favoured plate fixation for early and sustained functional recovery and lower rates of complications, although these studies focused on a single sub-type of distal radius fracture [11]. The authors observed variability in the populations studied, the numbers randomised, the treatments compared, and the outcome measures used, and they were unable to provide definitive treatment directives [11].

The heterogeneity in anatomical injury patterns in distal radius fractures creates considerable treatment challenges. Historically, fracture management objectives have focused on four elements: (1) restoration of anatomy; (2) stable fracture fixation; (3) preservation of blood supply; and (4) early mobilisation of the limb and the patient. More recently, the management of fractures has shifted to establishing a more holistic approach to the treatment of the fracture which considers the “personality” of the injury. This approach combines details of the fracture pattern, soft tissue injury and person characteristics [12], as these factors influence treatment decisions.

Recovery in many patients can be challenging [13] and can result in prolonged reduction in function and health related quality of life [14]. Reducing unwarranted variation in clinical care and the increased burden resulting from issues of both (mis)diagnosis and failure to follow evidence-based guidelines warrants a different approach [15]. An approach that considers the full spectrum characteristics of patients and combines details of the fracture pattern and soft tissue injury is needed.

### Clinical registries and Electronic Medical Records (EMRs)

Clinical quality registries (registries) have been used to inform clinical practice and health service decision making by way of routine prospective collection, analysis and reporting of health-related information [16, 17]. At the core of high-calibre registries is high quality, complete and valid data collected in a standardised way. The Australian Commission on Safety and Quality in Health Care’s (ACSQHC) operating principles for registries require limitation of core data collection to essential elements which are epidemiologically sound (i.e. simple, objective, reproducible, valid, and collected in a systematic manner across all contributing institutions). These guidelines were developed prior to widespread implementation of EMRs and the recent rapid evolution of artificial intelligence (AI). As a result, registries are predominantly populated by structured data with a focus either on readily accessible routine coding or extensive investment in the manual collection of data items.

Much of the data contained in the EMR and associated systems is *unstructured* in nature [18, 19]. Unstructured data includes text notes, written reports, and imaging. These data are often heterogenous, fragmented and not easily organised into a format that can be readily used by registries or incorporated into clinical algorithms and feedback loops. This type of data is often challenging [20], as it has no particular format or schema [21], and is often *noisy* [22]. However, unstructured data sources contain relevant, richly detailed, and nuanced data that could enhance the capability of registries to inform health care improvement. Recent advances in multi-modal deep learning algorithms that combine unstructured and structured EMR data have been found to improve the performance of prediction models and reduce errors [23]. Deep learning AI techniques that cater for both unstructured text and images in healthcare provide an opportunity to explore the ability to extract untapped information previously hidden [24] and provide insights into the fracture “personality”.

### Maximising the use of unstructured data

Registries have been used to identify patterns and associations and formulate hypotheses about cause-and-effect relationships [16, 17, 25]. Prediction modelling of registry data could have the potential to inform treatment pathways but richly detailed data to differentiate person, fracture, and treatment characteristics is needed. Routine administrative coding of diagnoses and procedures fails to provide sufficient phenotyping to develop robust prediction models capable of contributing to evidence-informed treatment pathways. In contrast, the unstructured data contained in surgical reports, operating theatre records, imaging reports and imaging contain rich data about fracture characteristics and management, including devices implanted. To date the unstructured nature of these data has precluded routine use of these data sources. Accessing these data in large, representative samples of distal fracture patients could provide critical information for improving outcome prediction.

In particular, orthopaedic trauma registries, such as the Victorian Orthopaedic Trauma Outcomes Registry (VOTOR), focus on collecting data about the patient (including their pre-injury function, health and employment), circumstances of the injury event, diagnoses, management and patient-centred and clinical outcomes [26–28]. These specific registries could provide an ideal platform to quantify the characteristics of the fracture injury and contribute to evidence-informed treatment [16, 17]. However, the barrier has been the lack of detail about fracture patterns (e.g. degree and location of comminution, degree of displacement, joint involvement, dorsal inclination, etc) and treatment due to reliance on administrative coding.

While detailed fracture classifications exist, these are rarely documented in medical records, and administrative diagnostic coding fails to characterise fracture details sufficiently [29]. In addition, there is classification error generated randomly, due to (lack of) inter-observer reliability, coding and transcription errors. The International Statistical Classification of Diseases and Related Health Problems, Tenth Revision, Australian Modification (ICD-10-AM) includes limited information about key characteristics of the fracture. For example, one third of distal radius fractures in VOTOR receive the “not further specified” code by hospital coders and laterality (i.e. left or right) is not captured at all. Similarly, routine coding of the management of fractures is done through Australian Classification of Health Interventions (ACHI) which provide a broad overview of the management such as the type of reduction of the fracture (closed vs. open) and whether fixation was used (but not the type of fixation).

Compounding issues with characterising fracture characteristics is the lack of barcode-to-patient tracking mechanisms or mandatory reporting of issues with most implants (e.g., plates, screws, wires). The majority of fracture implants are exempt from the Therapeutic Goods Administration requirement to provide patient information leaflets. The lack of routine and systematic post-market surveillance, and standardised capture of implant data at a patient-level places a greater reliance on collecting data about the detailed management of the fracture from other sources. Mining the heterogenous health data which already exists, with modern AI may offer the solution to unravelling previously hidden fracture details and characteristics.

### Artificial Intelligence (AI)

There are multiple examples where standard deep learning, such as Computer Vision and Natural Language Processing (NLP), has achieved outstanding performance in specific tasks (e.g., image/text classification, object detection, etc.) [19, 24, 30, 31]. Single model convolutional neural networks (CNN) deep learning has been used in studies to detect the presence or absence of distal radius fracture [32, 33], and has shown comparable performance to orthopaedic surgeons and superior performance to radiologists. Previous AI research has focused only on identifying the presence or absence of a fracture from a single source of information–either radiology reports or images. In contrast, humans often combine information from multiple modalities to acquire knowledge, with vision (images) and language (text) the most common signals used.

Inspired by how humans learn from multiple inputs, multimodal learning is becoming popular in AI, but remains under-explored in clinical research. The PRAISE study will develop a flexible and systematic multimodal deep learning system that reasons jointly on surgical reports, as well as pre- and post-operative imaging reports and imaging, to enable rich phenotyping of fracture type and care. This has the potential to enhance the capacity of registries to delineate fracture profiles, their treatment and outcomes, and provide valuable data for driving evidence-informed care to improve patient outcomes following a wrist fracture.

### Study aims

There are three aims of the PRAISE study:

1. Develop valid and reliable algorithms for identifying key distal radius fracture characteristics and treatment details using NLP and deep machine learning techniques applied to digital images, and surgical and radiology text reports.
2. Test whether AI derived fracture and treatment characteristics improve prediction of patient-reported outcome measures (PROMs) and clinical outcomes following distal radius fracture, compared to prediction models based on standard registry data.
3. Establish the documenting and supporting code for deployment of the AI algorithms into health data platforms to enhance clinical registry data collection and availability of key data to clinicians.

## Materials and methods

### Study design

The PRAISE study will be a multi-centre study of adult (16+ years) patients presenting to the emergency department (ED), treated in a short stay unit (SSU), or admitted to hospital for >24h for management of a wrist fracture in four Victorian hospitals. The study will use data from VOTOR, and data from the EMR, including X-rays, surgical reports and radiology reports.

### Inclusion and exclusion criteria

Participants will be eligible for this study if they meet the following criteria:

1. Date of injury July 2010 to June 2020;
2. Registered on the VOTOR with an ICD-10-AM diagnosis code pertaining to distal radius fracture **or** presentation and management of a distal fracture in the ED **or** management of a distal radius fracture SSU of the VOTOR participating hospitals; and
3. Aged 16 years or over at the time of presentation.

Patients presenting with a pathological fracture related to metastatic disease will be excluded. As VOTOR uses an opt-out method for inclusion, only eligible patients who have not opted-out of the registry will be included in this study. The current opt-out rate is <1.5% and, therefore, the coverage of eligible admitted cases will be high. A waiver of consent will be used to obtain the additional ED presentation and SSU admission cases, as well as the imaging and text reports for all cases.

### Ethics approval

The PRAISE project received ethical approved from participating sites through the Victorian State Single Ethical Review process (Project number VSM/73423) and will be conducted in compliance the NHMRC National Statement on ethical Conduct in Human Research (2007) and the Note for Guidance on Good Clinical Practice (CPMP/ICH-135/95). A waiver of informed consent was granted by the Human Research Ethics Committees.

### Data sources

The PRAISE study will use four data sources: 1) VOTOR registry patient data; 2) ED patient data; 3) SSU patient data; and 4) linked VOTOR patient data. Data sources 1) to 3) will be used to achieve study aim 1) and data source 4) will be used to achieve study aim 2).

#### 1) VOTOR registry data

The VOTOR registry is the largest and most comprehensive orthopaedic trauma outcomes registry worldwide and is compliant with the ACSQHC operating principles for clinical quality registries. For patients hospitalised >24h for their wrist fracture, cases will be identified through VOTOR using the relevant ICD-10-AM diagnosis codes for the admission. Demographic, injury event, injury diagnosis (ICD-10-AM), procedures (ACHI), in-hospital outcomes, limited implant data, X-ray images, text reports from radiology and surgery, and patient-reported outcome measures (PROMs) will be extracted for all eligible patients for this study. The PROMs are collected prospectively via telephone interview at 6-, 12- and 24-months post-injury [34], and include the 12-item World Health Organization Disability Assessment Schedule (WHODAS), 5-level EQ-5D (EQ-5D-5L), numerical rating scale for pain, pain location, return to work and work disability questions. Follow-up rates exceed 80% at each time point. Linkage with the Victorian Registry of Births, Deaths and Marriages (VBDM) is used to identify post-discharge deaths.

#### 2) ED and SSU data

Patients presenting to and discharged from the ED with wrist fracture will be identified from the presenting diagnosis in the EMR, while SSU cases will be identified by the ICD-10-AM diagnosis codes for the admission. Limited demographic information (age, gender, socioeconomic status, comorbidities, marital status and type of residence), injury event (coded cause, place, activity and intent, day of week and time of day of presentation, diagnosis (ICD-10-AM and text), and treatment classification, length of stay data and X-ray images and text reports from radiology and surgery will be collected.

#### 3) Linked VOTOR data

The VOTOR registry data and data generated from the AI models in aim 1) for VOTOR cases will be sent to the Centre for Victorian Data Linkage (CVDL) for linkage with the Victorian Admitted Episodes Dataset (VAED) and the VEMD to provide admissions and ED presentations for the two years prior, and all admissions and ED presentations in the two years following, the VOTOR admission. This linked dataset will provide a comprehensive dataset for the prediction models, and will contain detailed descriptors of the fracture including fracture pattern, soft tissue injury and treatment, as well as demographic details, pre-existing health conditions, injury event details, treating healthcare service, and associated injuries.

### Power calculations

A random sample of 517 patient sets of X-ray and radiology reports, proportional to fractures admitted to hospital with fractures managed in the ED or short stay unit, will be selected for classification by an orthopaedic surgeon, to quantify the agreement between the AI techniques and human assessment [35]. This is based on the diagnostic test with a null hypothesis of 0.7, alternative hypothesis of 0.8, power>0.8 and prevalence of dorsal displacement type fractures of 30% [36]. Class probabilities from the fracture classification will be generated and compared with orthopaedic surgeon assessment of the images and reports (gold standard). Key fracture words/phrases in text and regions in images, correlated with the predictions, will be highlighted with attention weights using a visualisation interface for surgeons to review the results generated by the models. We will retain 20% of the collected visual-linguistic fracture data pairs from training to test the proposed models. More than 6,000 VOTOR and 4,000 ED only cases will be available for the study, making the sample size more than sufficient to meet aim 2 of the study.

### Statistical plan

**Fig 1:**
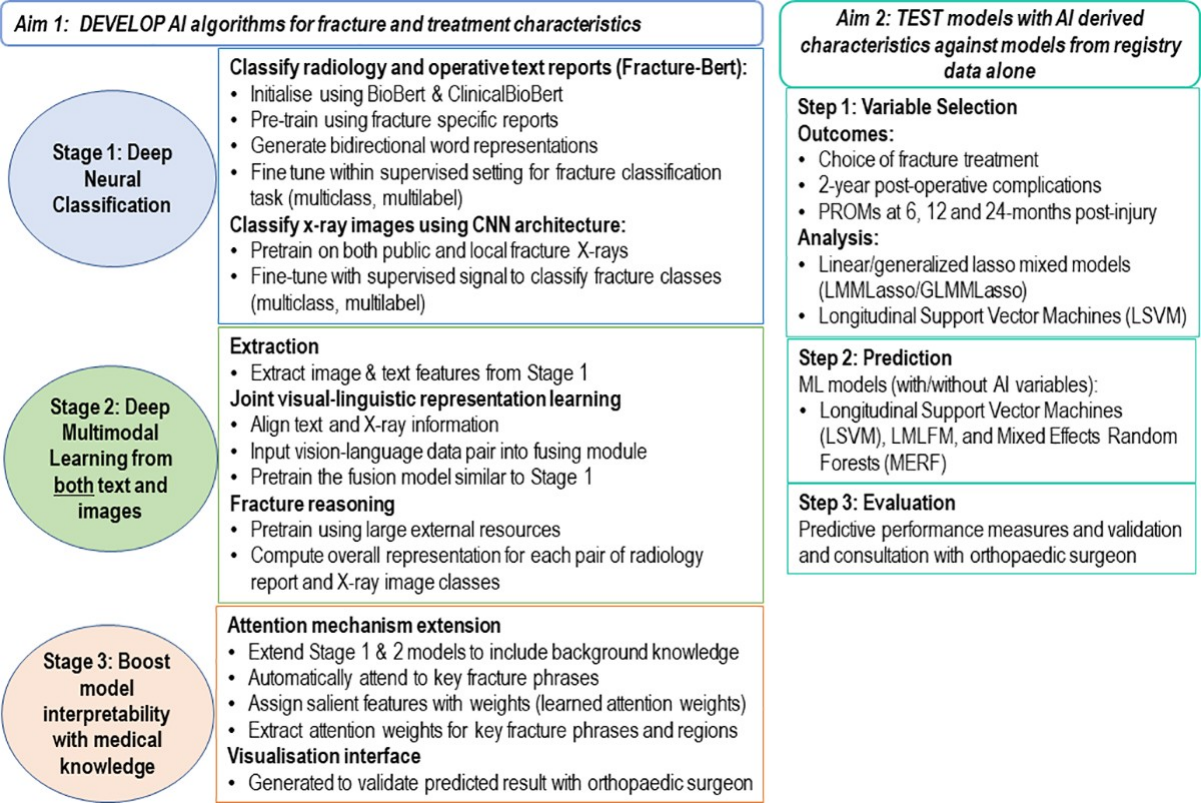


The analysis plan for the PRAISE study aims 1) and 2) is outlined in Fig 1.

### Aim 1: AI algorithms for fracture and treatment characteristics

For eligible ED, short stay unit and VOTOR registered distal radius fracture cases, the pre- and post-treatment X-ray images, radiology reports and surgical reports (where applicable) will be obtained for AI analysis. A multimodal deep learning fracture reasoning system will be developed that reasons jointly on X-rays and clinical text. The analysis will occur in three stages (Fig 1).

In *Stage 1*, we will develop specialised language representation models akin to Bert (Bidirectional Encoder Representations from Transformers) [37–39] called Fracture-Bert to generate bidirectional word representations that captures information related to specific distal radius fracture characteristics and treatment for the classification task. The X-ray images will be classified with deep CNN models [40, 41], where we pass each image through a series of fully-connected convolutional layers to learn features of various aspects of the X-ray image.

*Stage 2* will build a novel deep multimodal learning architecture that uses joint visual-linguistic representation learning from paired vision-linguistic data (i.e. paired medical images and radiology/operative text reports) to preserve and fuse semantics across modalities. Fracture reasoning will discriminate different fracture objects based on the learned latent representations. The pretrained architecture will use a variety of large external resources to complement the existing hospital data inputs and enhance the neural networks to improve predictions and advance learnings of fracture data.

*Stage 3* extends the models developed in Stages 1 and 2 to boost the model interpretability with background medical knowledge to highlight the key distal radius fracture features. The extended models with the attention mechanism [42, 43] will automatically attend to key phrases while predicting the corresponding fracture types. Salient fracture features will be assigned weights to generate the unimodal/multimodal representation. Learned attention weights will enhance our understanding of the key distal radius fracture characteristics and their treatment.

Fracture features will be categorised as present or absent based on the X-rays and radiology reports: radial height, radial inclination and ulnar variance; palmar inclination and teardrop angle; degree of comminution, etc. Treatment will be categorised into one of five categories: (1) closed reduction and immobilisation, (2) external fixation, (3) percutaneous wire fixation, (4) open reduction and internal fixation (plate), or (5) wrist fusion. Identification of surgical management and implant type from the X-ray images and operative report will be compared against the VOTOR implant data which is obtained from hospital ordering systems. Ordering systems currently are the most consistently available data source for orthopaedic trauma implants but the information extracted is highly variable, complete for only 60% of surgical cases, and does not specifically link implants to injury. VOTOR cases and ED presentation cases only will be compared for patient and fracture characteristics.

### Aim 2: Prediction modelling

The performance of models including the AI predicted indicators from the deep learning models will be tested against models including standard registry information using the linked VOTOR registry data (data source 4)). The capacity of the AI indicators to predict outcomes of clinical and post-operative complications, and PROMs post-injury. This will enable us to determine which radiological features based on the X-rays and text reports improve prediction over the standard data available in the registry. AI-derived fracture predicted probabilities and classes will be added to the VOTOR registry data and sent to the CVDL for the Linked VOTOR data. This linked dataset will provide a comprehensive longitudinal dataset for the prediction models.

The capacity of the AI derived fracture characteristics to predict outcome compared to the Linked VOTOR registry data alone will be tested using the following outcomes:

1. Fracture management complications: implant failure; non-union; mal-union of the fracture; infection; neurological injury
2. Readmission to hospital for fracture management
3. Presentation to the ED for fracture-related issues
4. Patient-reported outcomes at 6-, 12-, and 24-months after injury: EQ-5D-5L; 12-item WHODAS; pain scores; return to work and work-related disability.

A systematic approach to variable selection and model predictive performance will be implemented (Fig 1). Models will investigate predictive pathways and risk of outcomes of interest. State of the art longitudinal machine learning (ML) models [44] will be used for both the variable selection and prediction stages. The large number of covariates raise potential multicollinearity issues, and the possibility of unstable estimates. Therefore, where applicable a number of ML penalized regression methods will be implemented to reduce covariate numbers. The analyses will account for correlated and clustered responses to perform the variable reduction and prediction tasks.

Linear and generalized linear mixed models (LMMLasso/GLMMLasso) [45], longitudinal multi-level factorization machines model (LMLFMM) [46], longitudinal support vector regression (LS-SVM) [47] and mixed effects random forest (MERF) [48] will be used as these machine learning techniques can handle longitudinal data and a large number of potentially correlated features. The inclusion of predictions from Aim 1 will incorporate associated error that potentially compounds further error with their inclusion in the predictive models, so we will use several measures to review model performance. All models will be summarised by the predictive performance measures of accuracy, precision and recall [49] to select the best performing model for each outcome, and quantify the impact of the inclusion of the AI variables over registry data alone.

### Data security and confidentiality

Electronic data including images will be transferred from the participating sites to Monash University through a Secure File Transfer Protocol (SFTP) set up for this project. Once received at Monash, all data will be curated and analysed using the Monash Secure eResearch Platform (SeRP) and the Multimodal Australian ScienceS Imaging and Visualisation Environment (MASSIVE) platform. Monash SeRP is a secure environment for sharing research data for collaboration and analysis, within the control and governance of the data custodian. MASSIVE is a specialised high-performance computing facility for imaging and visualisation which has hardware dedicated to deep learning type applications (a pool of NVIDIA DGX1-V servers). Both SeRP and MASSIVE operate in ISO27001 compliant environments. Risks to patient privacy and confidentiality will be minimised through the use of specific study IDs and collated data will have identifiers removed. Only aggregate data will be presented.

## Discussion

Distal radius (wrist) fractures are the most common fracture presenting for medical care across all age groups. The anatomical pattern of injuries is diverse, there is widespread variation in clinical management, guidelines for management remain inconclusive, and the uptake of findings from clinical trials into routine practice has been limited. Robust predictive modelling which considers the characteristics of the fracture provides the best opportunity to inform care pathways and improve outcomes for patients. However, much of the data about the characteristics of the fracture is in unstructured data sources (x-ray images and text reports) which are not considered easily accessible and has made it difficult and restrictive for researchers to use in predictive modelling. This project will use existing data to assess the role of AI techniques to determine whether information gleaned from images and text reports can improve the prediction of clinical and longer-term patient reported outcomes following wrist fracture. Prediction models based on routinely collected registry data will be compared with models based on registry data and enhanced with additional information about fracture characteristics from the artificial intelligence techniques. The findings of this study have the potential to improve the automated collection of key information about fractures to support clinical decision making, guide personalised fracture care and improve outcomes for patients with wrist fractures by improving predictive models.

## Conclusion

In this study, the role of AI deep learning will be explored to determine whether AI techniques can improve the prediction of clinical and longer-term patient reported outcomes following distal radius fracture. The flexible three-stage multimodal deep learning fracture reasoning system will be used to extract important information from unstructured data sources including X-ray images, surgical and radiology text reports. Using existing data, prediction models with and without the AI enhanced findings from the deep learning system will be compared in order to enhance the capability of clinical registries to generate predictive analytics capable of guiding personalised fracture care.

## List of abbreviations

ACHI: Australian Classification of Health Interventions
ACSQHC: Australian Commission on Safety and Quality in Health Care
AI: Artificial Intelligence
BERT: Bidirectional Encoder Representations from Transformers
CNN: Convolutional Neural Network
DL: Deep Learning
DLFRS: Deep Learning Fracture Reasoning System
ED: Emergency Department
EMR: Electronic Medical Record
EQ-5D-5L: EuroQol- 5 Dimension 5-level
GLMM: Generalized linear mixed model
ICD-10-AM: International Classification of Diseases, 10th Revision, Australian Modification
LMM: Linear mixed models
LS-SVM: Longitudinal support vector regression
MERF: Mixed effects random forest
ML: Machine Learning
MLFMM: Longitudinal multi-level factorization machines model
NLP: Natural Language Processing
PROMs: Patient-reported outcome measures
SAIL: Secure Anonymised Information Linkage
SeRP: Secure eResearch Platform
TAC: Transport Accident Commission
UK: United Kingdom
US: United States
VAED: Victorian Admitted Episodes Dataset
VEMD: Victorian Emergency Minimum Dataset
VBDM: Victorian Registry of Births, Deaths and Marriages
VOTOR: Victorian Orthopaedic Trauma Outcomes Registry
WHODAS: World Health Organization Disability Assessment Schedule
